# Supplementary material for: MetaRibo-Seq measures translation in microbiomes
Source: Nat Commun. 2020 Jun 29;11:3268. doi: 10.1038/s41467-020-17081-z (PMC7324362; doi:10.1038/s41467-020-17081-z)
Supplement: Supplementary file 10 — Supplementary Data 7 [file 41467_2020_17081_MOESM10_ESM.zip › File2/Confidence_High_Taxonomy/109398_out.krona.html]

Javascript must be enabled to view this page.

members
magnitude
magnitudeUnassigned
count
unassigned
taxon
rank

109398\_out

25

2
25
superkingdom

25
phylum
1239

22
class
186801

species
1

SRS146813\_contig\_number\_contig-100\_1131.163600
2044939

186802
order
21

541000
6
family

2292180

SRS048870\_contig\_number\_contig-100\_106.139224SRS049959\_contig\_number\_649SRS049995\_contig\_number\_38497SRS150029\_contig\_number\_9193
species
4

1263
genus
2

species
2

SRS013476\_contig\_number\_41840SRS053398\_contig\_number\_9664
1264

1392389
15
genus


SRS015694\_contig\_number\_24256SRS052697\_contig\_number\_45297SRS054352\_contig\_number\_contig-100\_384.143428SRS055017\_contig\_number\_36803SRS075773\_contig\_number\_38784SRS078176\_contig\_number\_23970SRS078419\_contig\_number\_contig-100\_829.221270SRS098644\_contig\_number\_48878SRS1041032\_contig\_number\_15043SRS1041145\_contig\_number\_contig-100\_174.196192SRS104400\_contig\_number\_52896SRS142503\_contig\_number\_contig-100\_203.331797SRS144537\_contig\_number\_33979SRS147346\_contig\_number\_45986SRS893383\_contig\_number\_35740
1297617
15
species

1263003

SRS013521\_contig\_number\_contig-100\_1262.93726SRS053356\_contig\_number\_contig-100\_531.275497SRS062654\_contig\_number\_contig-100\_28221.70986
species
3
